# Vascular Endothelial Senescence: Pathobiological Insights, Emerging Long Noncoding RNA Targets, Challenges and Therapeutic Opportunities

**DOI:** 10.3389/fphys.2021.693067

**Published:** 2021-06-16

**Authors:** Xinghui Sun, Mark W. Feinberg

**Affiliations:** ^1^Department of Biochemistry, University of Nebraska–Lincoln, Lincoln, NE, United States; ^2^Nebraska Center for the Prevention of Obesity Diseases Through Dietary Molecules, University of Nebraska–Lincoln, Lincoln, NE, United States; ^3^Nebraska Center for Integrated Biomolecular Communication, University of Nebraska–Lincoln, Lincoln, NE, United States; ^4^Cardiovascular Division, Department of Medicine, Brigham and Women’s Hospital, Harvard Medical School, Boston, MA, United States

**Keywords:** cellular senescence, vascular endothelium, long noncoding RNAs, DNA damage, SASP, anti-senescent therapies

## Abstract

Cellular senescence is a stable form of cell cycle arrest in response to various stressors. While it serves as an endogenous pro-resolving mechanism, detrimental effects ensue when it is dysregulated. In this review, we introduce recent advances for cellular senescence and inflammaging, the underlying mechanisms for the reduction of nicotinamide adenine dinucleotide in tissues during aging, new knowledge learned from p16 reporter mice, and the development of machine learning algorithms in cellular senescence. We focus on pathobiological insights underlying cellular senescence of the vascular endothelium, a critical interface between blood and all tissues. Common causes and hallmarks of endothelial senescence are highlighted as well as recent advances in endothelial senescence. The regulation of cellular senescence involves multiple mechanistic layers involving chromatin, DNA, RNA, and protein levels. New targets are discussed including the roles of long noncoding RNAs in regulating endothelial cellular senescence. Emerging small molecules are highlighted that have anti-aging or anti-senescence effects in age-related diseases and impact homeostatic control of the vascular endothelium. Lastly, challenges and future directions are discussed including heterogeneity of endothelial cells and endothelial senescence, senescent markers and detection of senescent endothelial cells, evolutionary differences for immune surveillance in mice and humans, and long noncoding RNAs as therapeutic targets in attenuating cellular senescence. Accumulating studies indicate that cellular senescence is reversible. A better understanding of endothelial cellular senescence through lifestyle and pharmacological interventions holds promise to foster a new frontier in the management of cardiovascular disease risk.

## Introduction

Cellular senescence is a stable form of cell cycle arrest in response to various stressors. Senescent cells are characterized by a number of hallmarks, such as DNA damage, mitochondrial dysfunction, expression of cyclin-dependent kinase inhibitors and senescence-associated β-galactosidase (SA-β-gal), induction of the senescence-associated secretory phenotype (SASP), and changes in chromatin remodeling and metabolism (Munoz-Espin and Serrano, 2014; Sharpless and Sherr, 2015; Herranz and Gil, 2018; Hernandez-Segura et al., 2018; Gorgoulis et al., 2019; Khosla et al., 2020; Di Micco et al., 2021). It is increasingly recognized that cellular senescence is heterogeneous and cell-specific with diverse functions (Munoz-Espin and Serrano, 2014; Sharpless and Sherr, 2015; Lecot et al., 2016; Herranz and Gil, 2018; Hernandez-Segura et al., 2017, 2018, 2019; Gorgoulis et al., 2019; Khosla et al., 2020; Di Micco et al., 2021). Senescent cells are required for tissue remodeling and morphogenesis during embryonic development (Munoz-Espin et al., 2013; Storer et al., 2013), are essential for wound healing (Demaria et al., 2014), and promote heart regeneration after injury (Feng et al., 2019; Sarig et al., 2019). In contrast, senescent cells are found in obesity (Brodsky et al., 2004; Minamino et al., 2009; Wang et al., 2009; Ogrodnik et al., 2017), diabetes (Brodsky et al., 2004; Palmer et al., 2015), and atherosclerosis (Minamino et al., 2002; Childs et al., 2016), and contribute to the pathogenesis of these chronic and metabolic diseases. Targeted elimination of senescent cells in mice prevents neurodegeneration (Bussian et al., 2018), extends healthy life span (Baker et al., 2016), prevents type 1 diabetes (Thompson et al., 2019), and improves obesity-associated metabolic parameters (Palmer et al., 2019). To reconcile the beneficial and detrimental effects of cellular senescence, it was proposed that cellular senescence is an endogenous pro-resolving mechanism (Davan-Wetton et al., 2021). Controlled cellular senescence is important to maintain tissue homeostasis; however, when it is dysregulated leading to the over accumulation of senescent cells, cellular senescence triggers chronic inflammation and tissue damage contributing to the pathogenesis of various age-related diseases.

Long noncoding RNAs (lncRNAs) are an important class of RNAs lacking protein coding capacity and are longer than 200 nucleotides in length. Genomic studies have identified a number of disease-associated gene variants overlapping with lncRNA genes (Iyer et al., 2015; Dechamethakun and Muramatsu, 2017; Freedman et al., 2017; Hon et al., 2017; Giral et al., 2018; Castellanos-Rubio and Ghosh, 2019). Accumulating studies have identified a growing list of lncRNAs involved in human disease (Halim et al., 2017; Hennessy et al., 2019; Lyu et al., 2019). Recently, several lncRNAs have emerged as crucial regulators of cellular senescence in the vascular endothelium in mice (Hofmann et al., 2019; Shihabudeen Haider Ali et al., 2019; Bian et al., 2020; Haemmig et al., 2020).

The vascular endothelium, the innermost lining of blood vessels, is a critical interface between blood and all tissues. Emerging studies highlight that perturbation of endothelial cell (EC) senescence is a potential link between inflammation and aging. Here, we review the recent advances in our understanding of cellular senescence focusing on cellular senescence of the vascular endothelium, endothelial-leukocyte interactions, and how regulation by epigenetic mechanisms such as lncRNAs impacts the pathobiology in a range of aging-associated diseases. At the end, we discuss the therapeutic intervention, challenges and opportunities for targeting endothelial cellular senescence.

## Recent Advances in Cellular Senescence

### Cellular Senescence and Inflammaging

Inflammaging is a term describing the chronic low-grade sterile inflammation due to the impaired capacity of a host body to cope with stressors in the aging process (Franceschi et al., 2000). It underlies the increased risk of many diseases such as obesity, diabetes, and cardiovascular disease with age. The role of cellular senescence in inflammaging was first proposed about two decades ago (Franceschi et al., 2000), which was quickly accepted by other scientists (Butcher and Lord, 2004; Giunta, 2008). The number of senescent cells that increase dramatically in aging mice and humans likely results from at least one of two reasons. First, the accumulative effects of repetitive stresses increase the number of senescent cells during aging. Second, the impaired immune surveillance system cannot efficiently eliminate senescent cells with increasing age. These accumulated senescent cells secret a number of inflammatory cytokines and mediators that contribute to inflammaging. For example, CD4+ T cells from normoglycemic older subjects display a diabetes-associated Th17 profile that contributes to inflammaging (Bharath et al., 2020). These CD4+ T cells have defects in autophagy, mitochondrial function, and redox homeostasis (Bharath et al., 2020). Metformin alleviates inflammaging by enhancing autophagy and normalizing mitochondrial function in CD4+ T cells (Bharath et al., 2020).

Reciprocally, inflammation also induces cellular senescence, thereby contributing to an amplifying effect (Desdin-Mico et al., 2020). For example, metabolic failure of T cells causes the accumulation of cytokines in circulation (Desdin-Mico et al., 2020). This inflammatory status resembles inflammaging, and acts as a systemic inducer of cellular senescence. The premature aging in mice induced by T cell metabolic failure can be prevented by blocking tumor necrosis factor-α signaling and restoring nicotinamide adenine dinucleotide (NAD+) levels.

As evidenced by the above examples (Bharath et al., 2020; Desdin-Mico et al., 2020), inflammaging is targetable. Improving our understanding of the relationship between cellular senescence and inflammaging will likely provide new therapeutic targets to treat age-related disease by inhibiting the vicious cycle between cellular senescence and inflammaging.

### Cellular Senescence Reduces the Levels of NAD+ in Tissues During Aging

It is well-known that the levels of NAD+ decrease in our bodies during aging. The proposed underlying molecular basis involves the infiltration of macrophages in response to SASP factors elaborated by senescent cells. The levels of NAD+ decrease in part due to the increased expression and activity of CD38 during aging (Camacho-Pereira et al., 2016; Tarrago et al., 2018). CD38 is a NAD+-consuming enzyme that decreases the levels of tissue NAD+ through its ecto-enzymatic activity. The SASP factors secreted from senescent cells such as senescent ECs can increase the expression of CD38 in non-senescent cells *in vitro* (Chini et al., 2019). Recently, studies from two independent groups (Chini et al., 2020; Covarrubias et al., 2020) revealed that CD38+ proinflammatory macrophages are the major cells that cause the decline of NAD+ levels in white adipose tissue and liver of mice. The expression of CD38 is induced in the proinflammatory macrophages through the SASP factors of senescent cells *in vivo*. These studies uncovered a new causal link among senescent cells, CD38+ proinflammatory macrophages, and NAD+ decline in mice during aging. The levels of NAD+ also decline in vascular endothelium in aging, which leads to a decrease in blood flow of major organs whose function is critically dependent on blood flow (Das et al., 2018). A major question arose from this observation–does the aging-induced decrease of endothelial NAD+ result from impaired NAD+ biosynthesis or increased NAD+ consumption? ECs express NAD+ consuming enzymes including CD38, SIRT1, and poly(ADP-ribose) polymerases. In the cerebro-microvasculature, activated poly (ADP-ribose) polymerase is the main enzyme that utilizes NAD+ and contributes to the cognitive decline in mice (Tarantini et al., 2019). Future studies are needed to tease out the mechanisms that underlie the decline of NAD+ levels in the vascular endothelium in aging and disease.

### Cellular Senescence *in vivo*–Insights Learned From p16 Reporter Mice

There are different mouse models available to study the phenotypes of senescent cells *in vivo*. The p16LUC reporter mouse was generated in 2014 that harbors a knockin of the luciferase gene into the p16INK4a locus (Sorrentino et al., 2014). The p16-3MR (trimodality reporter) is a very complex reporter mouse that can be used for the detection of highly expressing p16^+^ senescent cells by luciferase activity, selective elimination with ganciclovir, and isolation by red fluorescent protein (Demaria et al., 2014). p16 tdTom mice carry a reporter allele with tandem-dimer Tomato (tdTomato) knocked into the endogenous p16INK4a locus (Liu J. Y. et al., 2019). Recently, *in vivo* dynamics and heterogeneity of senescent cells were examined using novel p16 reporter mice and single cell transcriptome analysis (Omori et al., 2020). In this mouse strain, a tamoxifen controlled Cre expression cassette was inserted into the endogenous Ink4a locus of the p16 gene without affecting p16 expression. This line of mice was then intercrossed with the tdTomato line to excise the stop cassette upstream of the tdTomato gene to allow the expression of tdTomato in the presence of Cre expression. The authors revealed several very interesting observations (Omori et al., 2020): (1) the majority of but not all cells with high p16 expression are senescent; (2) the number of senescent cells is about 1–3% in different organs when the mice are at the age of 1 year old; (3) the half-life of senescent cells are about 2.6–4.2 months for different organs; (4) gene ontology analysis of downregulated genes in senescent cells revealed that genes involved in protein catabolism, modification, and degradation are enriched; and (5) short-term (3 weeks) elimination of senescent cells reduces lipid droplet accumulation and alleviates inflammation in the liver of a mouse model of nonalcoholic steatohepatitis. In a different line of p16 reporter mice, a Cre expression cassette was inserted into the endogenous p16 genomic locus that allows for the constitutive expression of Cre, which can be used to label or eliminate p16-expessing cells (Grosse et al., 2020). With the elimination of p16-expressing cells when mice were young, some of them became increasingly sick with untreatable skin ulcerations or requiring euthanasia at the age of 1-year old. The highest number of senescent cells (EGFP positive cells in the mTmG reporter mice) was found in the liver and the majority of them are vascular ECs (Grosse et al., 2020), which is consistent with the results from the first line of new p16 reporter mice (Omori et al., 2020). Moreover, CD31-positive cells (representing ECs) also dominate among senescent cells in other organs, including in the heart and lung. The liver senescent ECs had increased expression of SASP markers, and underwent major metabolic changes in fructose/mannose, nucleotides, and glutathione metabolism. Surprisingly, endocytic capacity and detoxifying function are enhanced in senescent liver sinusoidal ECs from mice at the age of 1-year old. Age-induced pseudo-capillarization eventually impairs both functions later in life that in turn contributes negatively to lifespan (Grosse et al., 2020). Continuous long-term removal of senescent liver sinusoidal ECs causes liver perivascular fibrosis because the removed cells are not replaced with new ECs. Acute elimination of senescent cells in old mice also results in a significant decrease of CD31-positive cells in the liver and other tissues which leads to an increase in blood-vessel permeability and subsequent perivascular fibrosis. These data demonstrate cell-specific effects associated with elimination of senescent cells. In particular, senescent liver sinusoidal ECs are structurally and functionally distinct; the removal of them can lead to detrimental consequences due to the activation of a fibrotic response in the absence of the replacement of these removed ECs (Grosse et al., 2020). Future studies will be needed to clarify what underlies the discrepancy in the consequences of eliminating senescent cells between the two studies described above. This also raises the possibility that selective senolytics may need to be designed to eliminate a specific population of senescent cells in different organs or at different senescent stages to avoid adverse consequences.

### Machine Learning in Cellular Senescence

Deep learning is a subfield of machine learning that employs biology-inspired neural networks to learn and model the complicated associations between data and output for classification and prediction (Tang et al., 2019). It has been studied for many different applications, such as cancer diagnostics (Kleppe et al., 2021), medical image analysis (Wiestler and Menze, 2020), multi-omics and big data analyses (Krassowski et al., 2020; Haemmig et al., 2021; Mahmud et al., 2021), long noncoding RNA research (Alam et al., 2020), and protein structural modeling and design (Gao et al., 2020). Recently, deep learning was employed to identify senescent ECs (Kusumoto et al., 2021). The authors developed a quantitative scoring system to examine cellular senescence of ECs. This Deep Learning-Based Senescence Scoring System by Morphology was also used to screen and identify compounds that suppress the senescent phenotypes. Moreover, meta-analysis was performed to study EC senescence utilizing eight published datasets of transcriptome analysis (Park and Kim, 2021). Then Meta-analytic-based machine learning analysis was used to identify common features of EC senescence in genes and pathways. The authors identified 36 core features of genes (top five genes are IGFBP5, IFI27, PLAT, MX1, and IFIT1), 57 core features of pathways (e.g., glycine-serine and threonine metabolism and phosphoglycerate dehydrogenase), and 13 SASP genes that are EC-specific (e.g., PLAT, PLAU, ICAM1, MMP1, FAS, IGFBP7, SERPINE1, TIMP2, KITLG, VEGFA, TIMP1, CCL8, and TNFRSF1A). Of note, the data inputs of the study are from different types of ECs and sources. The identified SASP genes might not be simultaneously activated in a specific type of EC senescence. As more scientists will feed their experimental data into computer models, machine learning and deep learning will yield many more surprises in the field of cellular senescence. It would be anticipated that a “digital” senescent cell could be produced in the future.

## Cellular Senescence of Endothelial Cells

Early studies have demonstrated that EC senescence occurs *in vivo*. Senescent ECs are detected in animal models and human tissues in many different patho-physiological conditions. For example, single and double balloon denudations of rabbit carotid arteries induces EC senescence revealed by SA-β-gal staining (Fenton et al., 2001), a commonly used marker of cellular senescence. The authors postulated that vascular cell senescence may contribute to atherogenesis and postangioplasty re-stenosis. Senescent ECs were also observed in Zucker diabetic rats (Chen et al., 2002; Brodsky et al., 2004). The number of senescent ECs increased sixfold at the age of 22 weeks old, which is associated with a dramatic induction of p53, p21, and p16 (Brodsky et al., 2004). These β-gal-positive ECs can be reduced by treatment with ebselen, a peroxynitrite scavenger (Brodsky et al., 2004). Recently, single-cell RNA sequencing revealed that about 10% of cerebromicrovascular ECs undergo cellular senescence in the brain of 28-months-old mice (Kiss et al., 2020). SA-β-gal-positive ECs are also present in human atherosclerotic plaques of aorta and coronary arteries (Vasile et al., 2001; Minamino et al., 2002), and adipose tissues of obese human subjects (Villaret et al., 2010). Senescent ECs exhibit a number of structural and functional changes, have more protein aggregation than young cells (Hwang et al., 2019; Kopacz et al., 2020), and display pro-inflammatory, pro-thrombotic, vasoconstrictive phenotypes, thereby promoting age-associated diseases (Jia et al., 2019).

One of the intriguing features of EC senescence is the changes in metabolism, which is not well-understood (Sabbatinelli et al., 2019). In quiescent ECs, glycolysis generates up to 85% of the total cellular ATP content (De Bock et al., 2013; Eelen et al., 2018; Rohlenova et al., 2018); and the primary function of mitochondria is to serve as a biosynthetic and signaling hub (Quintero et al., 2006; Groschner et al., 2012; Diebold et al., 2019) rather than to produce ATP. In general, senescent cells demand more energy from glycolysis. Senescent ECs seem to display a different senescence-associated metabolic shift. Studies revealed that senescent ECs have a decline in glycolysis (Unterluggauer et al., 2008; Kuosmanen et al., 2018), or in mitochondria-mediated oxidative phosphorylation (Kim Y. M. et al., 2018; Haemmig et al., 2020; Cheng et al., 2021). So what could be the energy source for senescent programming and SASP in ECs? It turns out that glutaminolysis substantially contributes to energy regeneration in senescent HUVECs (Unterluggauer et al., 2008); senescent HUVECs (late passage with 90% beta-gal positivity) exhibit very high glutamine consumption rates leading to more glutamate and lactate production, which is not sufficient to retain the levels of ATP in these cells. Interestingly, inhibition of glutaminase, the first enzyme within the glutaminolytic pathway, induces cellular senescence in early passage of HUVECs. Recently, it was found that glutaminolysis is required for the survival of senescent cells *in vitro* and *in vivo*–the inhibition of glutaminolysis eliminated senescent cells and ameliorated age-associated organ dysfunction (Johmura et al., 2021). Examination of glutaminolysis on EC metabolism under different disease states may offer new druggable targets to control EC senescence.

### Common Stressors That Cause Endothelial Cell Senescence

#### DNA Damage

The DNA damage response resulting from genotoxic, oxidative, and metabolic stress is controlled by three phosphoinositide 3-kinase-related kinases: Ataxia-telangiectasia mutated (ATM), ATM- and Rad3-related, and DNA-dependent protein kinase (Shimizu et al., 2014; Blackford and Jackson, 2017). The DNA damage response orchestrates the appropriate repair of DNA damage coordinating with other ongoing cellular processes such as proliferation, cell survival, apoptosis, and senescence. p53-mediated signaling downstream of ATM is one of the key branches of the DNA damage response (Shiloh and Ziv, 2013), which plays important roles in cellular senescence. In response to DNA damage, cGAS-STING signaling is also important in promoting cellular senescence (Sun and Harris, 2020). Human primary ECs are more sensitive to genotoxic substances such as beno[a]pyrene, a component in cigarette smoke and a common mutagen in the environment than human primary smooth muscle cells, and pericytes, because nucleotide excision repair proteins Excision repair cross complementing-group 1 (ERCC1), XPF, and ligase I are expressed at lower levels in human primary ECs in culture.

Are ECs also more sensitive to genotoxic substances *in vivo*? Unrepaired DNA damage contributes importantly to vascular aging and the development of cardiovascular disease (Durik et al., 2012; Bautista-Nino et al., 2016; Uryga et al., 2016; Shah et al., 2018). ERCC1 is a mammalian endonuclease that is required for both nucleotide excision repair and Fanconi anemia inter-strand crosslink repair. ERCC1 also prevents endogenous DNA damage through an uncharacterized mechanism (Mulderrig and Garaycoechea, 2020). ERCC1-deficiency in mice is also a mouse model of accelerated aging. Its deficiency leads to cellular senescence in cells and mice (Kim et al., 2020) and is dependent upon ATM kinase (Zhao et al., 2020). ATM is activated in senescent cells in culture and tissues from ERCC1-deficient mice and naturally aged mice. Genetic and pharmacologic inhibition of ATM attenuates senescent phenotypes in ERCC1-deficient cells and mice. Recently, mice with EC-specific loss of ERCC1 were generated (Bautista-Nino et al., 2020). These mice died at the age of 5.5–6 months. At the age of 5 months, these mice developed an increased permeability of the renal microvasculature. Aorta and iliac arteries of ERCC1 EC-specific knockout mice exhibited decreased endothelium-dependent relaxations in response to acetylcholine, which is associated with reduced NO availability and increased oxidative stress. Endothelium-dependent relaxation was also impaired in coronary arteries. ERCC1 EC-specific knockout mice showed a hypertrophic aorta wall with outward remodeling. These data demonstrated that endothelial genomic instability can cause vascular aging and macrovascular and microvascular dysfunction.

#### Mitochondrial Dysfunction

Mitochondrial dysfunction in ECs can cause cellular senescence (Schleicher et al., 2008). Mitochondria are dynamic organelles and their function is maintained through proper coordination of mitochondrial biogenesis, dynamics (fission and fusion), and turnover (mitophagy). Alterations in mitochondrial morphology and function in ECs have been observed in obesity and diabetes (Shenouda et al., 2011; Costa et al., 2016). For example, increased mitochondrial fission was observed in venous ECs freshly isolated from patients with diabetes mellitus (Shenouda et al., 2011). Defects in mitochondrial function play a key role in the regulation of cellular senescence through different mechanisms such as mitochondria-borne reactive oxygen species (Ziegler et al., 2015; Wiley et al., 2016; Chapman et al., 2019; Vasileiou et al., 2019), though a recent study raised the question whether mitochondria are the main source of reactive oxygen species in the cell (Pak et al., 2020). In comparison to young HUVECs, old senescent HUVECs have extended and interconnected mitochondria, which is associated with the reduced expression of Fis1 and Drp1, two important proteins involved in mitochondrial fission (Mai et al., 2010; Lin et al., 2015). Silencing of Drp1 induces senescence in young HUVECs with increased SA-β-gal staining, elongated mitochondria, impaired autophagy, as well as increased p21 and p16 expression. Drp1 expression is also reduced in the endothelium of aorta in old rats associated with impaired autophagic processing. Drp1 knockdown also impairs autophagic flux in the vascular endothelium of common carotid arteries in rats (Lin et al., 2015). Excessive mitochondrial fission also induces endothelial senescence (Kim Y. M. et al., 2018). Loss of protein disulfide isomerase A1 causes EC senescence through inducing mitochondrial fragmentation and mitochondrial reactive oxygen species production. The underlying molecular basis is that knockdown of protein disulfide isomerase A1 results in mitochondrial fragmentation by increasing Drp1 sulfenylation at cysteine 644 and in turn Drp1 activity (Kim Y. M. et al., 2018).

#### Disturbed Flow

The vascular endothelium is exposed to different mechanical stimuli that can be detected and converted into biochemical signals and responses through mechanoreceptor proteins (Jufri et al., 2015; Xu et al., 2018), mechanosensitive transcription factors (Niu et al., 2019), other types of mechanosensors (Fang et al., 2019), and endothelial glycocalyx (Dragovich et al., 2016; Moore et al., 2021). Disturbed flow is one type of mechanical stimuli that occurs in arterial bifurcations and curvatures, areas where atherosclerosis develops with the presence of senescent ECs (Chiu and Chien, 2011; Warboys et al., 2014). It plays important roles in regulating gene expression, metabolic changes, endothelial function, atherogenic pathways, and EC fates such as cellular senescence (Wu et al., 2017; Demos et al., 2020; Dominic et al., 2020; Nakayama et al., 2020). The molecular mechanisms by which disturbed flow promotes endothelial senescence are incompletely characterized (Dominic et al., 2020). First, disturbed flow activates the DNA damage response. It has been demonstrated that disturbed flow induces EC senescence via a p53-p21 signaling pathway, which is attenuated by sirtuin 1 activation (Warboys et al., 2014). Moreover, disturbed flow causes telomere dysfunction. Disturbed blood flow induces the phosphorylation of telomeric repeat-binding factor 2 (TERF2)-interacting protein and subsequent nuclear export of its complexes with TERF2 *in vitro* and *in vivo*, instigating atherosclerosis (Kotla et al., 2019b). EC-specific knockout of TERF2-interacting protein inhibited disturbed flow-induced EC senescence and atherosclerotic plaque formation. In a separate study, Makorin-1 was identified as a major downstream molecule of TERF2-interacting protein (Kotla et al., 2019a). Makorin-1 is a transcriptional co-regulator and an ubiquitin E3 ligase. Phosphorylated TERF2-interacting protein reduced the expression of makorin-1, which is critical for EC senescence and SASP induced by disturbed flow (Kotla et al., 2019a). Although the molecular mechanism downstream of makorin-1 was not studied yet, these results indicate that protein posttranslational modification and proteasome-dependent degradation are likely important players in EC senescence and SASP. Lastly, disturbed flow induces the production of excessive reactive oxygen species (Heo et al., 2011). These three primary mechanisms are interconnected. While neither the mechanosensors nor the endothelial glycocalyx have been examined for the underlying mechanisms linked to senescence, it has been speculated that impaired structure and function of endothelial glycocalyx could mediate the adverse effects of disturbed flow and lead to cellular senescence. This is in part because disturbed flow leads to the degradation of endothelial glycocalyx (Mensah et al., 2020) and the thickness and barrier function of the endothelial glycocalyx decreases in aging (Machin et al., 2018).

#### Oxidative Stress

Hydrogen peroxide is widely used to induce cellular senescence in cell culture. While it is a signaling mediator under physiological conditions, it is also a main form of endogenous reactive oxygen species that can induce oxidative stress and cellular senescence *in vivo*. The main sources of hydrogen peroxide are NAPDH oxidase, xanthine-oxido-reductase, endothelial nitric oxide synthase, and mitochondrial respiration complexes in vascular endothelium (Cai, 2005; Breton-Romero and Lamas, 2014). The levels of hydrogen peroxide also increase in vascular endothelium resulting from impaired antioxidant machinery such as endothelial loss of thioredoxin reductase 2 (Kirsch et al., 2016). When hydrogen peroxide levels exceed the antioxidant capacity of cells, it induces cellular senescence through different mechanisms. In late-passage ECs, the levels of intracellular reactive oxygen species increase which contribute to cellular senescence by accelerating telomere shortening (Haendeler et al., 2004; Kurz et al., 2004). Interestingly, this is mediated by inducing the nuclear export of telomerase reverse transcriptase into the cytosol (Haendeler et al., 2004). Early studies indicated that oxidative stress rather than telomere shortening plays a dominant role in inducing cellular senescence of ECs isolated from atherosclerotic chronic smokers (Farhat et al., 2008). Oxidative stress causes oxidative DNA, lipid, and protein damage, all can promote cellular senescence of vascular endothelium. It should be noted that the exogenously added hydrogen peroxide in many studies does not mimic the more selective spatiotemporal pattern of endogenous hydrogen peroxide flux, even its concentration is much higher than endogenous hydrogen peroxide, and has different functional consequences (Saeedi Saravi et al., 2020).

#### Telomere Shortening

Inhibition of telomere function induces senescence, while the introduction of telomerase restores the impaired function associated with senescence in human aortic ECs (Minamino et al., 2002). In coronary ECs obtained from 11 patients with coronary artery disease, telomere length is shorter than in age-matched patients without coronary artery disease. In addition, the telomere length is also shorter in cells at atherosclerotic lesions than at the same location of non-atherosclerotic lesions (Ogami et al., 2004). When the telomere reaches a critical length, it activates the DNA damage response (d’Adda di Fagagna et al., 2003; Takai et al., 2003), thereby promoting cellular senescence. As discussed above, oxidative stress and disturbed flow can promote cellular senescence, at least in part, by accelerating telomere shortening.

#### Radiation

Radiation-induced vascular injury is commonly observed in cancer patients after radiation therapy (Murphy et al., 2012; Venkatesulu et al., 2019; Ramadan et al., 2021). The major drivers of radiation-induced vascular injury are oxidative stress, DNA damage, and inflammation (Lafargue et al., 2017; Baselet et al., 2019). The vascular endothelium is likely more susceptible to radiation-induced cellular senescence (Aratani et al., 2018; Venkatesulu et al., 2018) and contributes to the higher rates of radiation-induced cardiovascular disease in cancer patients (Wang et al., 2016). Chronic low-dose levels of gamma radiation accelerates endothelial senescence as revealed by an increase in SA-β-gal staining (Yentrapalli et al., 2013a). The expression of a great number of radiation-induced proteins are altered, including an increase in p53, p21, and plasminogen activator inhibitor-1 (PAI-1) expression. These irradiated cells also display increased oxidative stress, decreased nitric oxide availability, and impaired PI3K-Akt signaling (Yentrapalli et al., 2013a,b; Azimzadeh et al., 2015). Radiation-induced endothelial senescence was also observed in rat cerebromicrovascular ECs in response to a higher amount of gamma-irradiation (2–8 Gy) (Ungvari et al., 2013). The gamma-irradiated cells expressed a higher level of p16 expression and acquired SASP characterized by an increased expression of proinflammatory cytokines and chemokines, including IL-6, IL-1α, IL-8, and monocyte chemotactic protein-1 (Ungvari et al., 2013). A large number of proteins were altered in their expression in the secretome of senescent ECs induced by irradiation. This irradiation-induced SASP affects the function of nonirradiated neighbor cells in a STAT3- and ICAM-1-mediated mechanism (Philipp et al., 2017). In response to radiation, endothelial function is impaired with the following features (Venkatesulu et al., 2018; Baselet et al., 2019): (1) ECs manifest a sterile pro-inflammatory state resulting from DNA damage and oxidative stress; (2) endothelium-mediated vasodilation is impaired; (3) ECs display a pro-coagulant and pro-thrombotic phenotype; (4) ECs exhibit mitochondrial dysfunction and metabolic perturbations; (5) radiation induces EC death; and (6) ECs become senescent.

**FIGURE 1:**
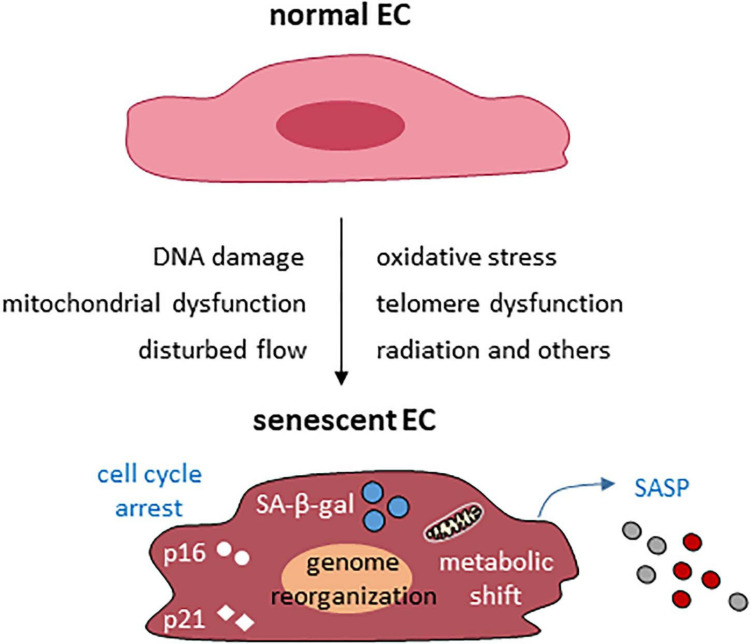
Common causes and features of endothelial cellular senescence. EC, endothelial cell.

The common causes and features of endothelial cellular senescence are summarized in Figure 1. In addition to the common causes of cellular senescence of the vascular endothelium, amyloid β 1-42 oligomer (Donnini et al., 2010; Singh Angom et al., 2019), hyperglycemia (Prattichizzo et al., 2018), oxidized LDL, homocysteine (Zhang et al., 2015), and angiotensin II (Yang D. et al., 2019; Khemais-Benkhiat et al., 2020) all can induce or accelerate endothelial cellular senescence.

### Recent Advances in Endothelial Cell Senescence

In the last several years, a number of new mechanisms and regulators such as lncRNAs (discussed in Section “Long Noncoding RNAs in Cellular Senescence of Vascular Endothelium” below) were identified in EC senescence (Liu B. et al., 2019; Pan et al., 2021). Here, we discuss recent progress in EC senescence focusing on *in vivo* studies within the last 3 years unless otherwise being discussed in other sections.

Loss of sirtuin activities is involved in the pathogenesis of cardiovascular and metabolic disease (Kane and Sinclair, 2018). Here, we discuss several recent studies about the role of sirtuins in cellular senescence of the vascular endothelium. SIRT1 is a member of the sirtuin family consisting of seven histone deacetylases requiring NAD+ for enzymatic activity. EC-specific SIRT1 knockout mice exhibit a decrease in blood flow and endurance with age (Das et al., 2018). In contrast, overexpression of endothelial SIRT1 has a protective effect. NAD+ repletion rescued the age-associated decrease in capillary density and blood flow, an effect that can be boosted by hydrogen sulfide (Das et al., 2018). The protective effects of SIRT1 on vascular aging is mediated, at least in part, by inhibiting the expression of PAI-1. PAI-1 was first identified as a marker and mediator of cellular senescence in fibroblasts (Kortlever et al., 2006; Vaughan et al., 2017). PAI-1 is a master contributor of homocysteine-induced endothelial senescence and vascular aging. Pharmacological inhibition of PAI-1 blunts homocysteine-induced cellular senescence of ECs in culture and in mice, indicating that PAI-1 is a potential target to improve endothelial and cardiac health (Sun et al., 2019). Inhibition of PAI-1 expression by SIRT1 mediates in part the protective effects of SIRT1 in vascular endothelial senescence (Wan et al., 2014). SIRT1 is inversely correlated with PAI-1 in senescent HUVECs, aortas of aged mice, and human atherosclerotic plaques. Furthermore, SIRT1 inhibition increases PAI-1 expression in young HUVECs, and conversely, SIRT1 overexpression decreases PAI-1 expression in senescent HUVECs. Importantly, EC-specific transgenic SIRT1 overexpression decreased PAI-1 expression in the aortas of old mice mediated by binding to the PAI-1 promoter and reducing the acetylation of histone H4 lysine 16 (Wan et al., 2014).

Another family member, SIRT6, also protects ECs from senescence. Its expression is decreased in senescent ECs and EC-specific deletion of SIRT6 exacerbated vascular aging through forkhead box M1, a critical transcription factor for cell cycle progression and senescence (Lee et al., 2020). These studies demonstrate that SIRT6 has a protective role in the aging vasculature (Lee et al., 2020). In a separate study, EC-specific deletion of SIRT6 enhanced blood pressure by impairing endothelial function, whereas SIRT6 overexpression ameliorated EC senescence (Guo et al., 2019). Mechanistically, SIRT6 prevented hypertension by inducing the expression of GATA-binding protein 5 (GATA5) through inhibiting the expression of NK3 homeobox 2, a transcriptional repressor, by deacetylating histone H3 lysine 9 in its promoter (Guo et al., 2019). The potential role of GATA5 and NK3 homeobox 2 in regulating EC senescence was not examined in this study (Guo et al., 2019). Recently, it was found that SIRT7 expression was reduced in the vascular endothelium of a mouse model of progeria–a premature aging syndrome (Sun et al., 2020). EC-specific restoration of SIRT7 by recombinant adeno-associated virus serotype 1 ameliorated aging features and extended life span in progeria mice (Sun et al., 2020). These new studies provide additional evidence for EC-specific sirtuin-activating therapies in treating and preventing aging and aging-related cardiovascular and metabolic diseases.

Angiopoietin-like 2 promotes vascular inflammation, endothelial dysfunction, and atherosclerosis (Farhat et al., 2013; Horio et al., 2014). In support of this, knockdown of angiopoietin like-2 by a small hairpin RNA mediated by adeno-associated virus serotype 1 delayed the formation of atherosclerotic plaques and reduced cellular senescence and inflammation in the aortic endothelium (Caland et al., 2019). One week after the knockdown of angiopoietin like-2, the number of senescent ECs was reduced due to apoptosis. Four weeks post knockdown, the expression of endothelial progenitor markers was increased in the endothelium suggesting endothelial repair. In arteries of atherosclerotic patients, the expression of angiopoietin like-2 strongly correlates with p21 expression. These data suggest that therapeutic down-regulation of angiopoietin like-2 may be protective for atherosclerosis by eliminating senescent ECs and stimulating endothelial repair.

CD9 is an important regulator of senescence in ECs. Its expression is increased in arteries of older humans and in the atherosclerotic plaques in humans and mice (Cho et al., 2020; Kim and Choi, 2020). Knockdown of CD9 in senescent ECs attenuates senescence, and its overexpression in young ECs promotes senescence. CD9 neutralization or ablation decreased the formation of atherosclerotic lesions in *ApoE^–/–^* mice. These data indicate that CD9 is a potential target for preventing and treating vascular aging and atherosclerosis. CD9 is highly expressed in leukocytes (Reyes et al., 2018). Future studies will need to clarify whether leukocyte-derived extracellular vesicles containing CD9 contribute to the increase of CD9 in ECs during aging.

Cdc42, a member of the Rho GTPase family, is involved in the regulation of actin cytoskeleton and serves as a focal node of various signaling pathways in cells. Endothelial deletion of CDC42 reduced pro-inflammatory markers, endothelial senescence, and atherosclerotic lesion formation in mice (Ito et al., 2014). Knockdown of cdc-42 in mutant worms restored the shortened lifespan highlighting an evolutionary conserved role for this protein in aging.

The role of senescent ECs was examined recently in diabetic retinopathy (Crespo-Garcia et al., 2021). Senescent ECs accumulate in retinas during peak pathological neovascularization in a mouse model of ischemic retinopathy and of patients with diabetic retinopathy. Small molecule inhibitors of the anti-apoptotic protein BCL-xL selectively eliminated a population of senescent ECs by inducing apoptosis, and ameliorated oxygen-induced retinopathy. These studies demonstrate that senescent ECs are central to pathological retinal angiogenesis and they rely on BCL-xL for survival. The broad implication is the potential use of BCL-xL inhibitors for eliminating senescent ECs in other diseases including cardiovascular and metabolic diseases.

### SASP in ECs

Senescent cells actively produce a complex secretome known as the SASP (Coppe et al., 2010). An “SASP Atlas” was recently identified using unbiased proteomic profiling in several types of senescent cells induced by different stimuli (Basisty et al., 2020). The authors found that soluble SASP (as relative to exosome SASP; see below) protein components of the same cell type are highly heterogeneous and inducer-specific. In addition, the soluble SASP protein components are also largely distinct for each cell type. However, 17 soluble SASP proteins are shared by all cell types and inducers examined. Similarly, exosomes/extracellular vesicles-resident SASP proteins are largely distinct for each senescent phenotype. These data demonstrate that protein components of SASP is highly complex and dynamic in a cell type- and inducer-specific manner. Future studies are required to examine the RNA and lipid components of SASP as our knowledge about them is limited. Below we discuss what we know about SASP of ECs.

Senescent ECs can produce different SASP phenotypes depending on the type, duration, and magnitude of stresses, and their growth environment. HUVECs with replicative senescence produce high levels of interleukin 8 (IL-8) (Hampel et al., 2006), IL-6, PAI-1, and monocyte chemotactic protein-1 (Lin et al., 2015). Senescent HUVECs induced by irradiation or doxorubicin produce high levels of C-X-C motif chemokine 11 (Hwang et al., 2020). In senescent HUVECs induced by disturbed flow, cells display a strong SASP phenotype including excessive ROS, which is different from the SASP produced by HUVECs with replicative senescence (Dominic et al., 2020). IL-1α was recently identified as an important functional component of SASP in both replicative and premature senescent HUVECs (Barinda et al., 2020). Chronic TNF-α exposure promotes the SASP characterized by high levels of adhesion molecules such as E-selectin and ICAM1 (intercellular adhesion molecule 1), cytokines including IL-6 and IL-8, as well as PAI-1 and IGFBP-5 (insulin-like growth factor binding protein-5) in HUVECs (Khan et al., 2017). The dominant and functional SASP factors are different in these *in vitro* studies. This is probably also determined by epigenetic mechanisms (see Section “Long Noncoding RNAs in Cellular Senescence of Vascular Endothelium” below). The studies about SASP of ECs *in vivo* are scarce. In older adults, a panel of seven SASP factors were detected in human plasma, that associate with age, frailty, and adverse post-surgery outcomes (Schafer et al., 2020). Circulating SASP factors such as GDF15 (growth differentiation factor 15), osteopontin, and IL-8 are abundantly produced and secreted by senescent EC and their circulating levels increase with aging. The complete catalog of EC SASP factors needs to be created in the future using quantitative proteome analysis. Circulating SASP factors may serve as clinically useful biomarkers of age-related diseases to predict disease risk.

Senescent ECs produce more functional extracellular vesicles that exert autocrine or paracrine effects. These extracellular vesicles are classified as small extracellular vesicles (Riquelme et al., 2020), microvesicles (Carracedo et al., 2019), or microparticles (Abbas et al., 2017), and are thought to represent different subpopulations of extracellular vesicles. They can promote cell migration (Riquelme et al., 2020) and impact the function of other vascular cells leading to the development of cardiovascular disease (Carracedo et al., 2019). Microparticles from senescent ECs or patients with acute coronary syndromes promote premature EC aging under atheroprone low shear stress and thrombogenicity through angiotensin II activated signaling (Abbas et al., 2017). Extracellular vesicles can mediate the communication between ECs and vascular smooth muscle cells (Hergenreider et al., 2012). EC-derived extracellular vesicles induced protein synthesis and senescence in vascular smooth muscle cells in culture (Boyer et al., 2020). It is known that extracellular vesicles contain non-coding RNAs, such as microRNAs and lncRNAs (Hergenreider et al., 2012; Khalaj et al., 2019). EC-derived extracellular vesicles can regulate gene expression in recipient cells through these microRNAs and lncRNAs. Thus, EC-derived extracellular vesicles are important SASP components that warrant additional attention.

SASP can mediate many patho-physiological effects of senescent cells (Gorgoulis et al., 2019; Birch and Gil, 2020). It is well known that senescent ECs can affect the function of vascular smooth muscle cells in the vessel wall due to reduced nitric oxide bioavailability. Moreover, senescent ECs are sufficient to impair insulin signaling in mice consuming normal chow diet through a SASP factor - IL-1α (Barinda et al., 2020). Furthermore, the SASP of ECs induced by irradiation or doxorubicin can adversely affect the success of cancer therapy (Hwang et al., 2020). Lastly, the SASP factors secreted by senescent ECs trigger platelet activation *in vitro* (Venturini et al., 2020). Although it was not revealed how the involved SASP factors mediated these effects, it would be interesting to identify them and examine their roles *in vivo*. Improving our understanding of SASP of senescent ECs will add more tools in our arsenal to combat aging-related diseases by tailoring SASP, which is of importance when elimination of senescent cells could result in adverse effects.

### Sex Differences in Vascular Endothelial Senescence

Sex differences in cardiovascular aging and age-related diseases have been observed in mice and humans (Merz and Cheng, 2016; DuPont et al., 2021), which can result from changes in the expression of sex hormones, hormone receptors, and other steroid receptors such as mineralocorticoid receptors during aging. As an example, sex-specific mechanisms regulate arterial stiffness, a pathological state defined as resistance to deformation or a loss of elastic compliance caused by changes in the geometry and microstructure of the vascular wall (Ogola et al., 2018; DuPont et al., 2019). Arterial stiffness occurs naturally in aging and accelerated by chronic metabolic disease states. The causal role of endothelial senescence in arterial stiffness is reviewed recently (Jia et al., 2019). One of the major underlying mechanisms is that the impaired NO bioavailability in senescent ECs promotes arterial stiffness (Ogola et al., 2018; Jia et al., 2019). However, our knowledge about sex differences in endothelial cellular senescence is very limited. An age-related increase in arterial telomere uncapping and senescence is greater in women than men (Walker et al., 2016), indicating a sex difference in vascular senescence. Future studies should reveal the key players, mechanisms, and signaling pathways about it in both preclinical animal models and human subjects including postmenopausal females and age-matched males.

## Long Noncoding Rnas in Cellular Senescence of Vascular Endothelium

**FIGURE 2:**
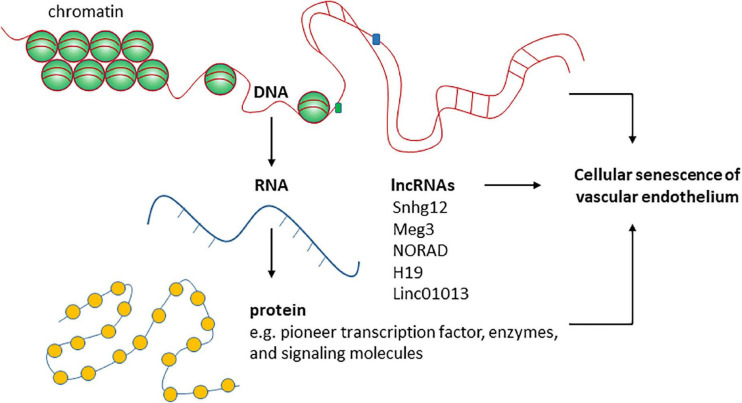
The roles of functional RNA molecules in regulating endothelial cellular senescence are not well- studied.

The regulation of cellular senescence involves different layers of mechanisms at chromatin, DNA, RNA, and protein levels (Figure 2). The main epigenetic mechanisms including DNA methylation, histone modifications, and regulation by non-coding RNAs are all important in regulating cellular senescence. Cellular senescence is associated with many changes in chromatin structure and function (Criscione et al., 2016). Chromatin accessibility is redistributed in HUVECs during senescence, which is determined in part by the AP-1 transcription factor family such as ATF3 (Zhang et al., 2021). Senescence-associated changes in DNA methylation primarily involve global hypomethylation and focal hypermethylation (Yang and Sen, 2018). Hypomethylation occurs particularly at repeat regions, while focal hypermethylation occurs at CpG rich promoter sequences (Yang and Sen, 2018). Histone methylation is an important mechanism controlling EC senescence by regulating gene expression (Yang et al., 2020). Specifically, Smyd3, a histone H3 lysine 4 methyltransferase, induces the expression of PARP16 [poly(ADP-ribose) polymerase 16] by increasing the levels of histone H3 lysine 4 trimethylation at the promoter region of PARP16 gene, an endoplasmic reticulum membrane protein that promotes cellular senescence of rat aortic ECs by increasing endoplasmic reticulum stress in response to angiotensin II. Recently, KAT7, a histone acetyltransferase, was identified as a key driver of cellular senescence in human mesenchymal precursor cells (Wang et al., 2021). KAT7 increases histone H3 lysine 14 acetylation, activates p15^*INK4b*^ transcription, and induces cellular senescence (Wang et al., 2021). It remains unclear if KAT7 is an important driver of cellular senescence by inducing the expression of genes that promote cellular senescence in other cell types such as ECs?

Three-dimensional genome organization is an early event on the path to senescence of different cell types including ECs (Zirkel et al., 2018). This is determined by DNA binding proteins such as high-mobility group box protein 2 (HMGB2) and CCCTC-binding factor. Both are key players in regulating genome structure by determining interchromosomal and intrachromosomal interactions (Aird et al., 2016; Lazniewski et al., 2019; Wang et al., 2019). HMGB2 expression was depleted from cell nuclei upon cellular senescence. The depletion alters genome organization by inducing a heterochromatic shift characterized by alterations in histone methylation and acetylation and inducing spatial CCCTC-binding factor clustering. These changes in genome structure allows the entry of senescence. Relevant points to this process include: (1) the intrachromosomal interactions of the same chromosomal regions are cell-type-specific upon senescence entry; and (2) at this stage on the path to senescence, the senescent program is likely reversible. The remaining questions are: how does the genome reorganization reprogram gene expression to inhibit the expression of genes involved in cell proliferation while inducing the expression of genes involved in SASP? Is the structure of genomic loci containing SASP genes maintained to allow active gene expression? How does the genome reorganization coordinate with key signaling pathways such as p53 signaling and cGAS-STING signaling that promote cellular senescence? Are lncRNAs important in determining the functional output of the genome during genome reorganization upon senescence entry? With advanced techniques such as ATAC-seq, chromatin conformation capture (3C) and related approaches become available to more scientists, these questions will be answered and new knowledge about the role of genome structure and chromatin modification in cellular senescence will be revealed. Below we describe the role of lncRNAs in regulating cellular senescence of the vascular endothelium.

### Small Nucleolar Host Gene-12

**FIGURE 3:**
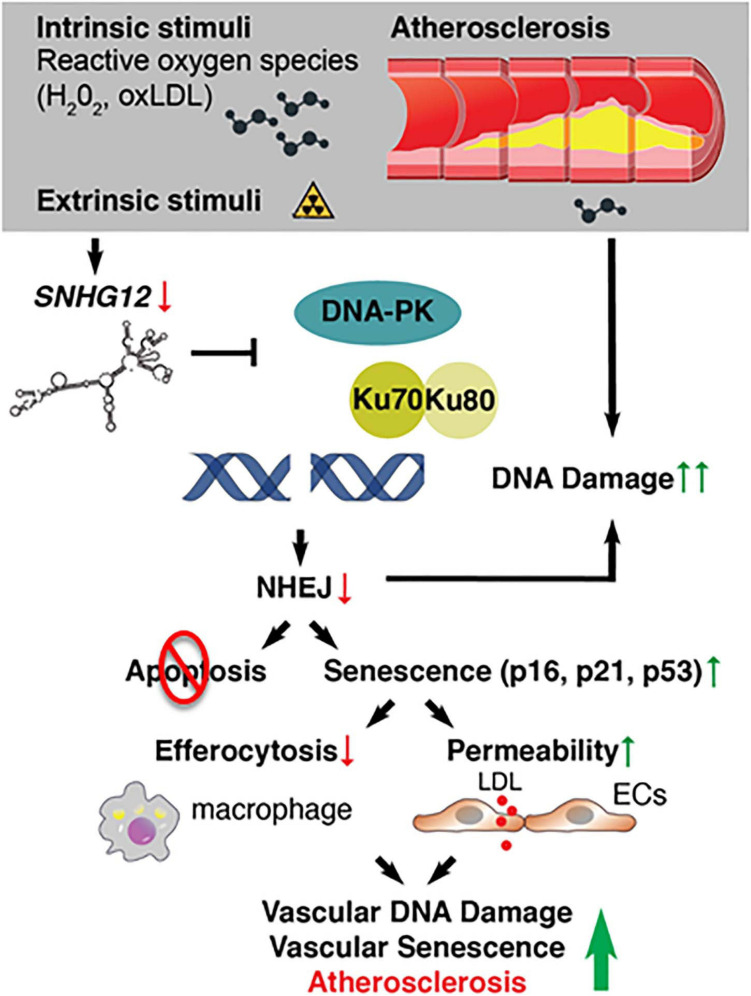
Deficiency of long noncoding RNAs (IncRNA) SNHG12 triggers DNA double-strand breaks, endothelial senescence, and atherosclerosis.

Small nucleolar host gene-12 (*Snhg12*) regulates angiogenesis, tumor cell growth, apoptosis, and immune escape (Wang et al., 2017; Zhao et al., 2018). We recently identified this lncRNA as an important regulator of DNA damage response in the vessel wall (Haemmig et al., 2020). RNA-sequencing of the aortic intima in *Ldlr*^–/–^ mice revealed that the lncRNA *Snhg12* was significantly downregulated during the progression phase of atherosclerosis (Haemmig et al., 2020). Mechanistically, *Snhg12* positively regulated DNA repair machinery through a direct lncRNA-protein interaction with DNA-PK and, in turn, potently suppressed cellular senescence (Figure 3). Reduced DNA damage in the vascular endothelium as a result of *Snhg12* overexpression prevented vascular senescence, LDL transcytosis (permeability to LDL), and atherosclerotic lesion formation. In contrast, biweekly intravenous injections of LNA gapmer *Snhg12* achieved nearly 50% reduction in *Snhg12* expression in the intima and increased atherosclerotic lesion size (Haemmig et al., 2020). These findings highlight that dynamic alteration of lncRNA-protein interactions in the vascular endothelium can impact the DNA damage response, endothelial senescence, and vascular disease.

The protective role of *Snhg12* in the vessel wall was also revealed by a separate study (Qian et al., 2021). *Snhg12* expression was also reduced by Ang II in HUVECs. The overexpression of *Snhg12* inhibited EC senescence *in vitro* through a miR−25−3p/SIRT6 pathway and alleviated vascular endothelial injury in Ang II−induced hypertensive mice (Qian et al., 2021).

### Maternally Expressed Gene 3

**FIGURE 4:**
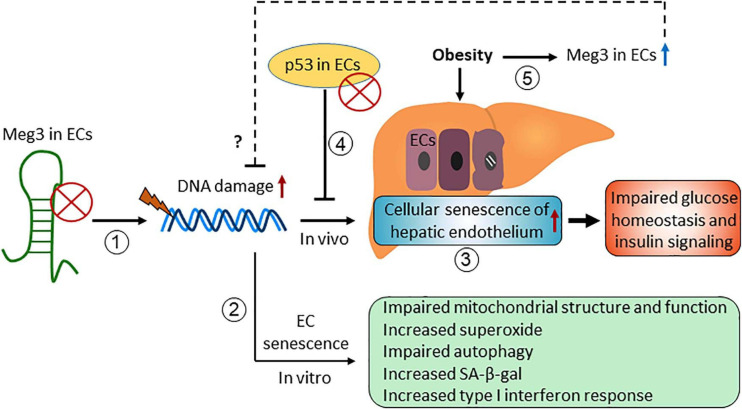
The role of maternally expressed gene 3 (Meg3) in regulating endothelial cellular senescence.

Maternally Expressed Gene 3 (Meg3) was first identified as a non-protein coding gene in mice (Schuster-Gossler et al., 1998). It received considerable attention since it was revealed that Meg3 is one of the top 15 high expressing lncRNAs in HUVECs in 2014 (Michalik et al., 2014). Meg3 functions in many different biological processes through its interaction with different binding partners, including several proteins such as p53, EZH2, PTBP1, and PTBP3 (Zhao et al., 2010; Zhu J. et al., 2015; Zhang et al., 2017; Shihabudeen Haider Ali et al., 2019). Here, we summarize the role of Meg3 in cellular senescence (Figure 4).

Emerging evidence have demonstrated that Meg3 expression limits cellular senescence *in vitro* and *in vivo*. Meg3 expression is elevated in senescent HUVECs compared with early passage cells (Boon et al., 2016), and in HUVECs exposed to oxidative stress (Fuschi et al., 2017) and hypoxia (Neumann et al., 2018). We have shown that DNA damaging agents induce Meg3 expression in HUVECs in a p53-dependent manner, and Meg3 knockdown induces DNA damage and inhibits EC proliferation (Shihabudeen Haider Ali et al., 2019). These effects of Meg3 knockdown are mediated in part by its protein binding partner PTBP3. Meg3 knockdown causes cellular senescence of HUVECs characterized by accelerated telomere length shortening, an increase in the levels of superoxide, an increase in SA-β-gal activity, impaired autophagy, and mitochondrial dysfunction (Lan et al., 2019; Cheng et al., 2021). Moreover, we found that Meg3 deficiency promoted cellular senescence of the hepatic endothelium with no or minimal effects on cellular senescence of other cell types in the liver in diet-induced obese mice (Cheng et al., 2021). Importantly, Meg3 knockdown impaired systemic glucose homeostasis and insulin signaling in the liver, which can be restored by attenuating the cellular senescence of hepatic endothelium, indicating that cellular senescence of the vascular endothelium impairs glucose homeostasis and insulin signaling in obesity (Cheng et al., 2021). How Meg3 limits cellular senescence of ECs is unclear. We speculate that Meg3 is involved in DNA damage repair or promotes autophagy in response to different stresses (Shihabudeen Haider Ali et al., 2019; Cheng et al., 2021).

### NORAD

LncRNA noncoding RNA activated by DNA damage (NORAD) is also known as linc00657. The initial functional analysis revealed that NORAD maintains genomic stability (Lee et al., 2016). Norad-deficient mice undergo premature aging characterized by genomic instability and mitochondrial dysfunction (Kopp et al., 2019). NORAD interacts with proteins such as PUMILIO and RBMX, which likely mediate its effects on genomic stability (Munschauer et al., 2018; Elguindy et al., 2019). NORAD is highly expressed in ECs (Michalik et al., 2014), which is required to limit endothelial senescence (Bian et al., 2020). The expression of NORAD increases in aged HUVECs, and NORAD knockdown aggravated ox-LDL-induced cellular senescence revealed by the increased number of SA-β-gal-positive cells and the increased expression by p53 and p21 at the mRNA level (Bian et al., 2020). NORAD knockdown also increased the production of reactive oxygen species and lipid peroxidation, which likely mediates the effects of NORAD knockdown on cellular senescence (Bian et al., 2020). NORAD interacts with SFPQ (Bian et al., 2020), a ubiquitous nuclear RNA-binding protein involved in RNA biogenesis. The authors also showed that NORAD knockdown promoted the expression of proinflammatory molecules and the development of atherosclerosis in *ApoE^–/–^* mice (Bian et al., 2020). However, the causal relationship between cellular senescence of vascular endothelium and atherosclerotic lesion formation was not examined. Future studies are needed to identify the molecular basis by which NORAD knockdown induces cellular senescence in primary ECs.

### H19

H19 is one of the first lncRNAs discovered several decades ago (Brannan et al., 1990). H19 is an important regulator of cellular senescence in the vascular endothelium (Hofmann et al., 2019). H19 expression is reduced in the vascular endothelium of aged mice. Silencing of H19 leads to cell cycle arrest at the G0/G1 phase, increases the number of SA-β-gal positive cells and the expression of p16 and p21 in ECs. Moreover, H19 depletion impaired the sprouting capacity of ECs in an *ex vivo* aortic ring assays and reduced capillary density in the ischemic leg of mice subjected to hindlimb ischaemia. Furthermore, the depletion of H19 enhanced inflammatory responses indicating that the reduction in H19 expression in vascular endothelium contributes to inflammaging. Mechanistically, H19 suppresses STAT3 activity and the expression of STAT3 target genes to limit endothelial senescence and inflammaging. However, the role of H19 in regulating cellular senescence of vascular endothelium is unclear in atherosclerosis. H19 expression might promote it because H19 expression contributes to the development of atherosclerosis (Huang et al., 2019; Yang Y. et al., 2019). The role H19 in cellular senescence of hepatic endothelium is unknown, although H19 induces hepatic steatosis (Liu et al., 2018; Wang et al., 2020). Thus, the function of H19 in cellular senescence of the vascular endothelium can be protective or detrimental in a disease-specific manner.

### Linc01013

Linc01013 is also known as Age and EndMT regulated RNA in endothelium (Aerrie) (Pham et al., 2020). The function of linc01013 is poorly characterized. Recently, it was revealed that its expression was induced by oscillatory flow and aging in HUVECs, and induced in atherosclerotic plaques from symptomatic patients compared to asymptomatic patients and in ischemic heart tissues from the left ventricle (Pham et al., 2020). Loss of Aerrie impaired cellular migration, barrier function, and angiogenic sprouting of HUVECs (Pham et al., 2020). Aerrie interacts with Y-Box protein 1 (YBX1), a protein involved in stress response and DNA repair among other functions in mammalian cells (Pham et al., 2020). Loss of Aerrie leads to DNA damage and activated DNA damage signaling (Pham et al., 2020). Silencing YBX1 rescued DNA damage signaling activated by the loss of Aerrie without any effects on DNA damage itself (Pham et al., 2020). Aerrie overexpression did not affect DNA damage under basal conditions, while it reduced DNA damage induced by doxorubicin in HUVECs (Pham et al., 2020). These data demonstrated that lncRNA Aerrie and YBX1 and their interaction are important for DNA damage repair in ECs (Pham et al., 2020). It would be interesting to examine whether there is a mouse homolog for human lncRNA Aerrie, and whether the role of Aerrie in DNA damage operates in vascular endothelium in mice and humans.

## Anti-Senescent Therapies Targeting the Vascular Endothelium

Our understanding of cellular senescence has been greatly improved making it an attractive target of therapies. The class of pharmacological interventions targeting senescent cells, known as senotherapeutics, has garnered significant attention. Cellular senescence can be promoted, prevented, eliminated, modulated, and reverted depending on the disease context (Davan-Wetton et al., 2021). The pharmacological interventions that inhibit cellular senescence can be classified into senolytics, senomorphics, and senescent immunotherapy (Kim and Kim, 2019; von Kobbe, 2019; Song et al., 2020). Senolytics eliminate senescent cells by attenuating apoptosis evasion to induce their cell death; senomorphics disrupt key attributes of senescence such as by blocking the SASP (without killing senescent cells); and senescent immunotherapy clears senescent cells by harnessing immune cells (Kim and Kim, 2019; Song et al., 2020). Among many types of senotherapeutics (Niedernhofer and Robbins, 2018; Kim and Kim, 2019; von Kobbe, 2019; Song et al., 2020; Di Micco et al., 2021), we mainly discuss nicotinamide riboside (NR) and rapamycin as examples of senomorphics, and quercetin/dasatinib as an example of senolytics.

### Nicotinamide Riboside

Nicotinamide riboside (NR) is one of the most studied NAD+ precursors that holds promise of decreasing the body’s susceptibility to different aging-related diseases (Trammell et al., 2016). It can be used to restore the levels of NAD+ which steadily declines with age in multiple tissues including endothelium (Csiszar et al., 2019). NAD+ is a cofactor in many redox and nonredox reactions. Therefore, NR influences many cellular functions that are regulated by NAD+, including energy metabolism, DNA repair, cellular senescence, among others (Covarrubias et al., 2021). In animal studies, NR has shown beneficial effects in cardiovascular and metabolic diseases (Kane and Sinclair, 2018). For example, administration of NR in food fully attenuated the increases in DNA damage, endothelial senescence, and lesion formation mediated by *Snhg12* knockdown in the vessel wall in a mouse model of atherosclerosis (Haemmig et al., 2020). In a recent preclinical study, NR was shown to inhibit endothelial inflammation, promote NO-mediated vasodilation, and prevent Ang II-induced endothelial dysfunction (Mateuszuk et al., 2020). Atherosclerosis continue to claim millions of lives annually, and endothelial dysfunction and inflammation play an important role in the initiation and progression of atherosclerosis. Future studies exploring NR in combination with other goal-derived medical therapies (e.g., statins) will be of interest in the context of patients with coronary artery disease or at risk for myocardial infarction. Chronic supplementation with NR appears to be well-tolerated in healthy middle-aged and older adults, which effectively stimulates NAD+ metabolism (Martens et al., 2018). Several clinical trials aiming to improve cardiovascular and metabolic diseases have been completed using NR as a dietary supplement. While NR supplementation alone had no beneficial effects on insulin sensitivity, mitochondrial function, energy metabolism, ectopic lipid accumulation, and plasma markers of inflammation in healthy overweight or obese individuals (Dollerup et al., 2018, 2020; Remie et al., 2020), there is evidence demonstrating that NR can reduce inflammaging in the elderly (Elhassan et al., 2019) and prevent tissue senescence and inflammation in aged mice (Desdin-Mico et al., 2020).

Several studies are currently underway to determine whether NR improves functioning in peripheral artery disease^1^ (Identifier NCT03743636), arterial stiffness and elevated systolic blood pressure in patients with moderate to severe chronic kidney disease (see footnote text 1; Identifier NCT04040959), and memory and brain blood flow in older adults with mild cognitive impairment (see footnote text 1; Identifier NCT03482167). Data from these trials will be essential to advance our understanding of the role of NR in improving cardiovascular health.

### Rapamycin

Rapamycin is FDA approved to prevent transplant rejection due to its immunosuppressive properties and is also used in drug-eluting stents to inhibit coronary artery restenosis. Accumulating studies over the past two decades demonstrate that rapamycin exhibits senomorphic properties. Mechanistically, it is known that rapamycin targets the activity of mTOR complex 1 (mTORC1) and mTOR complex 2 (mTORC2) (Kim and Guan, 2015), although the exact mechanisms underlying its senomorphic effects remain poorly understood in specific cell types. Rapamycin-mediated inhibition of the TOR pathway extends lifespan across several species from yeast to mice (Saxton and Sabatini, 2017) in part by delaying cellular senescence and suppression of the SASP. In ECs, rapamycin reduced cytokine or oxLDL-induced adhesion molecules and macrophage adhesion to ECs (Wang et al., 2014; Sun et al., 2018). However, another study showed that rapamycin induced endothelial-to-mesenchymal transition due to activation of autophagy and activation of the TGF-β pathway *in vitro* (Sasaki et al., 2020). Because higher doses of rapamycin typically used in transplant patients are associated with a 20% increased risk of a range of side effects such as mouth sores, rash, or hyperlipidemia, lower doses have been used in anti-aging clinical studies. In one cohort of cardiac rehabilitation patients, ∼60% experienced diarrhea (Singh et al., 2016). Another trial using rapamycin at 1 mg for 8–16 weeks in adults (*n* = 28) aged 70–95 years old with stable chronic disease was tolerated reasonably better compared to placebo with small decreases in hemoglobin, and only two participants developed either diarrhea or facial rash. Interestingly, there was no difference in circulating levels of SASP and surprisingly an increase in TNFα (Kraig et al., 2018). Collectively, these data highlight that long-term use of low-dose rapamycin may be required to uncover potential benefits. Because mTOR signaling is active in many cell types beyond the vascular endothelium such as in immune cells, careful attention to impact on innate immunity, susceptibility to sepsis, and cancer will be warranted for its use as a senomorphic therapeutic.

### Quercetin and Dasatinib

While dasatinib alone did not confer senolytic activity in ECs, a combination of dasatinib (D; the pan–tyrosine kinase inhibitor) and quercetin (Q; naturally occurring flavonoid) has been used to attenuate senescence in HUVECs (Zhu Y. et al., 2015), mice (Ogrodnik et al., 2017), and humans (Justice et al., 2019). In preclinical studies, senolytic treatment (D+Q) reduced the number of senescent cells in the vessel wall of chronologically aged mice, which improved relaxation of carotid arteries to acetylcholine (Roos et al., 2016). While in atherosclerotic mice, D+Q only reduced the number of senescent cells in the media and not in regions with established intimal atherosclerotic plaques, it improved NO signaling, and reduced intimal plaque calcification (Roos et al., 2016). Interestingly, quercetin alone decreased lipid deposition in the arterial lumina of *ApoE–* mice likely by inhibiting oxLDL-induced endothelial senescence (Jiang et al., 2020). In p16-Cre/R26-mTmG reporter mice, the senolytic combination of D+Q efficiently eliminated F4/80-positive cells with high p16 expression, but not CD31-positive cells with high p16 expression in the livers, indicating the senolytic combination is ineffective to remove senescent liver ECs in mice (Grosse et al., 2020).

Several clinical studies have been completed using D+Q. For example, the first-in-human open-label study revealed that the intermittent D+Q improved physical function in the 14 patients with idiopathic pulmonary fibrosis (Justice et al., 2019). Specifically, 6 min walk distance, 4 m gait speed, chair-stands, and short physical performance battery score were improved at 1 week following the completion of D+Q treatment. This study suggests that it is feasible to examine D+Q in larger randomized and controlled trials for idiopathic pulmonary fibrosis. In another open label phase I pilot study, the 3-days administration of D (100 mg) and Q (1,000 mg) reduced the burden of senescent cells in adipose tissue as well as the levels of circulating SASP factors in patients with diabetic kidney disease (Hickson et al., 2019). In addition to the completed studies, several planned or ongoing studies will be examining D+Q for Alzheimer’s disease and frailty in adult survivors of childhood cancer (see footnote text 1; identifiers NCT04063124, NCT04685590, and NCT04733534). Additional clinical studies were completed using quercetin such as on cerebral blood flow, blood sugar and blood vessel function in type 2 diabetes, hypertension and endothelial dysfunction (see footnote text 1; identifiers NCT01376011, NCT01839344, and NCT01691404), others are ongoing about coronary artery disease progression, glucose absorption in obesity and obesity with type 2 diabetes, and Fanconi Anemia (see footnote text 1; identifiers NCT03943459, NCT00065676, and NCT01720147). Because high doses of dasatinib alone is known to induce pleural effusions in patients, an effect potentially mediated by endothelial permeability (Phan et al., 2018), lower doses of dasatinib will continue to be warranted for clinical applications.

In addition to NR, rapamycin and D+Q, there are other compounds that can be used to attenuate cellular senescence. Among them are resveratrol (Kim E. N. et al., 2018; Breuss et al., 2019), metformin (Bharath et al., 2020; Kulkarni et al., 2020), an inhibitor of glutaminase 1 that inhibits glutaminolysis (Johmura et al., 2021), and β-hydroxybutyrate (Han et al., 2018, 2020). These senotherapeutics target all types of senescent cells with less or more efficacy, and they are not EC-specific. Until now, there are a few studies that examined the effects of senotherapy on senescent ECs. The effects of quercetin, rapamycin, and NR have been examined on senescent ECs as discussed above. How to target senescent ECs is still in its infancy. To ambitiously develop EC-specific senotherapeutics, we will need to identify EC-specific players, mechanisms, and features of cellular senescence, and EC-specific mechanisms by which senescent ECs are eliminated by the immune system. Alternatively, different strategies can be developed and optimized to improve the selectivity of senotherapy targeting senescent ECs (Childs et al., 2017). There is still much to learn about how best to target senescent ECs in aging and diseases.

## Challenges and Future Directions

### Heterogeneity of ECs and EC Senescence

ECs are present in all organs and have organ-specific properties. Recently, two single-cell sequencing studies greatly advance our understanding of unique signatures of organ-specific ECs in mice (Kalucka et al., 2020; Paik et al., 2020). ECs are also heterogeneous within an organ. For example, liver ECs are heterogeneous in both mice and humans revealed by single-cell transcriptome analysis (Ramachandran et al., 2019; Xiong et al., 2019; Omori et al., 2020). A main subpopulation of liver ECs are known as liver sinusoidal ECs that line the hepatic sinusoids. In addition, there are other types of liver ECs such as periportal and pericentral ECs in mice (Xiong et al., 2019; Omori et al., 2020) or hepatic artery, central vein, lymphatic ECs in humans (Ramachandran et al., 2019). Most of different subpopulations of liver ECs express common endothelial markers such as CD31 (PECAM1) and VE-cadherin at different levels (Ramachandran et al., 2019; Grosse et al., 2020; Kalucka et al., 2020). However, different subpopulations of liver ECs also have differently molecular signatures and function. Because of the heterogeneity of ECs, EC senescence are likely very heterogeneous in chromatin modification, genome reorganization, gene expression and markers, SASP, life cycle dynamics, fates, functional consequences, and response to senolytics. With the increasing power and growing performance of single cell sequencing, much more will be learned about the heterogeneity of EC senescence particularly in vessel wall and liver that provide new insight into the pathogenesis of cardiovascular and metabolic diseases such as atherosclerosis and fatty liver disease.

### Senescent Markers and Detection of Senescent ECs

Great efforts have been made to identify robust markers of senescence (Hernandez-Segura et al., 2017; Aliper et al., 2019; Casella et al., 2019; Wei et al., 2019). Transcriptome analysis revealed that 68 RNA molecules are differentially expressed in senescent ECs and fibroblasts induced by different stressors. Some of them are non-protein coding RNAs (Casella et al., 2019). The novel and unique transcriptome signatures can be used to detect and differentiate different senescent phenotypes (Hernandez-Segura et al., 2017). Senescent markers are also needed to identify senescent ECs at different stages of their life cycle.

In addition to novel senescent markers, novel methods to detect senescent ECs *in vivo* are also needed. Several fluorescent probes have been developed to visualize senescent cells *in vivo* by detecting the activity of β–gal (Lozano-Torres et al., 2017; Chen et al., 2020). For example, a small molecule-based near-infrared fluorescent probe (BOD-L-βGal) was developed to detect β-gal (Chen et al., 2020). When the probes were incorporated into polymer nanoparticles, it can be used for the imaging and visualization of senescent cells in atherosclerotic arteries of mice (Chen et al., 2020).

Recently, a quantitative approach was established to identify and characterize senescent cells in aging and disease (Biran et al., 2017; Gal et al., 2019). The new approach requires the specific instrument that is the ImageStreamX imaging flow cytometer. It combines the quantitative power of flow cytometry with high content image analysis. It detects SA-β-gal activity using the bright field channel and additional senescence related markers such as p16 via multiple fluorescence channels (Gal et al., 2019). This also help identify the specific cell origin of senescent cells including ECs (Gal et al., 2019).

### Differences Between Mouse and Human in Immune System

There are significant differences in the immune system between mice and humans (Mestas and Hughes, 2004). The cells that are involved in the immune surveillance of cellular senescence are also different. For example, natural killer cells have different inhibitory receptors, cell surface markers (e.g., the expression of CD56 in human but not mouse natural killer cells), subsets, and functions (Murphy et al., 2012). In addition, these cells are likely exposed to different pathogens over the course of theirs lives in mice and humans (Tao and Reese, 2017). Given immunosenescence is important in regulating cellular senescence, a reassessment of cellular senescence in the vascular endothelium using a humanized immune system mouse models will be of interest (Allen et al., 2019).

### LncRNAs as Therapeutic Target

While numerous pre-clinical studies have investigated lncRNAs as therapeutic targets, translating these findings to viable therapeutics remains challenging, although significant progress has been made. For example, unprotected nucleic acids in the circulation are rapidly metabolized by the liver and kidneys. Even when novel delivery platforms such as lipid-based formulations and nanoparticles are utilized to improve pharmacokinetic properties of oligonucleotide therapeutics, targeting oligo-based therapies to diseased tissues remains a challenge to the field of lncRNA therapeutics. While high levels of hepatotoxicity were observed with the first generation of LNA gapmers in both pre-clinical studies and phase I clinical trials (Swayze et al., 2007; Burdick et al., 2014; Migawa et al., 2019; Shen et al., 2019), emerging chemistries such as cEt gapmers exhibit improved toxicity profiles (Seth et al., 2009, 2010). Emerging cell-specific delivery platforms such as peptide nucleic acids (PNAs) may facilitate targeting in tissues beyond the liver or in response to pathobiological stimuli. Future studies that modulate lncRNA-expressing loci using CRISPR-Cas9 or other gene editing methods in combination with viral, LNPs, or nanoparticle-based delivery platforms will be of interest. Finally, unlike other classes of noncoding RNAs such as microRNAs, lncRNAs are often thousands of base pairs long, making packaging and delivery to target tissues difficult. Overcoming these challenges will enable the translation of accumulating studies that have demonstrating the importance of lncRNAs in endothelial senescence to meaningful therapeutics and clinical trials.

## Author Contributions

Both authors contributed to the article and approved the submitted version.

## Conflict of Interest

The authors declare that the research was conducted in the absence of any commercial or financial relationships that could be construed as a potential conflict of interest.

## Footnotes

1 https://clinicaltrials.gov/

## Abbreviations

SA- β -gal: senescence-associated β -galactosidase
SASP: senescence-associated secretory phenotype
lncRNAs: long noncoding RNAs
ECs: endothelial cells
NAD+: nicotinamide adenine dinucleotide
ERCC1: excision repair cross complementing-group 1
TERF2: telomeric repeat-binding factor 2
HMGB2: high-mobility group box protein 2
*Snhg12*: small nucleolar host gene-12
Meg3: maternally expressed gene 3
NORAD: lncRNA noncoding RNA activated by DNA damage
Aerrie: age and endMT regulated RNA in endothelium
mTORC1: mTOR complex 1.
